# Estimation methods with ordered exposure subject to measurement error and missingness in semi-ecological design

**DOI:** 10.1186/1471-2288-12-135

**Published:** 2012-09-04

**Authors:** Hyang-Mi Kim, Chul Gyu Park, Martie van Tongeren, Igor Burstyn

**Affiliations:** 1 Department of Mathematics and Statistics, University of Calgary, Calgary, Canada; 2 School of Mathematics and Statistics, Carleton University, Ottawa, Canada; 3 Centre for Human Exposure Science, Institute of Occupational Medicine, Edinburgh, UK; 4 Department of Environmental and Occupational Health, Drexel University, Philadelphia, USA

**Keywords:** Constrained estimation, EM algorithm, Group-based strategy, Isotonic regression, Measurement errors

## Abstract

**Background:**

In epidemiological studies, it is often not possible to measure accurately exposures of participants even if their response variable can be measured without error. When there are several groups of subjects, occupational epidemiologists employ group-based strategy (GBS) for exposure assessment to reduce bias due to measurement errors: individuals of a group/job within study sample are assigned commonly to the sample mean of exposure measurements from their group in evaluating the effect of exposure on the response. Therefore, exposure is estimated on an ecological level while health outcomes are ascertained for each subject. Such study design leads to negligible bias in risk estimates when group means are estimated from ‘large’ samples. However, in many cases, only a small number of observations are available to estimate the group means, and this causes bias in the observed exposure-disease association. Also, the analysis in a semi-ecological design may involve exposure data with the majority missing and the rest observed with measurement errors and complete response data collected with ascertainment.

**Methods:**

In workplaces groups/jobs are naturally ordered and this could be incorporated in estimation procedure by constrained estimation methods together with the expectation and maximization (EM) algorithms for regression models having measurement error and missing values. Four methods were compared by a simulation study: naive complete-case analysis, GBS, the constrained GBS (CGBS), and the constrained expectation and maximization (CEM). We illustrated the methods in the analysis of decline in lung function due to exposures to carbon black.

**Results:**

Naive and GBS approaches were shown to be inadequate when the number of exposure measurements is too small to accurately estimate group means. The CEM method appears to be best among them when within each exposure group at least a ’moderate’ number of individuals have their exposures observed with error. However, compared with CEM, CGBS is easier to implement and has more desirable bias-reducing properties in the presence of substantial proportions of missing exposure data.

**Conclusion:**

The CGBS approach could be useful for estimating exposure-disease association in semi-ecological studies when the true group means are ordered and the number of measured exposures in each group is small. These findings have important implication for cost-effective design of semi-ecological studies because they enable investigators to more reliably estimate exposure-disease associations with smaller exposure measurement campaign than with the analytical methods that were historically employed.

## Background

Measurement error problems are common in a variety of fields. Long-term blood pressure, nutrient intake, or concentration of pollutants are just some example of variables [1]. Measurement errors should be taken into account in any inferential procedure. In fact, exposure measurement error causes bias of parameter estimates and loss of power for detecting relationships among variables [2]. In epidemiological cohort studies of occupational and environmental exposures, individual exposures are measured with errors and often not available for all members of the study, while health outcome measures are obtained for all individuals without measurement error. In such settings, a commonly employed approach is to derive exposure estimates via a group-based strategy (GBS, also known as semi-individual or semi-ecological study design). Individuals are grouped according to shared attributes, such as job title or work area, and assigned an exposure score, usually the mean of some concentration measurements made on samples drawn from the group. This amounts to single imputation of missing value, and is commonly used for all the individuals in the group [3].

In some settings the use of a group-based strategy for assigning exposure scores can result in a less biased estimate of an exposure-disease association than the case of using individual exposures measured with errors. However, it is often the case that there are only a few exposures measured in each group. Unlike the case with large number of observations in each group, the GBS incurs severe bias to the estimation.

Very often, groups of subjects are formed according to the level of a certain attribute. Then, it is reasonable to assume that the groups are also ordered in the same (or opposite) way. This information could be incorporated in inference for more precise analysis [4]. In order to incorporate the group ordering information, we use a constrained method and thereby achieve an improvement in estimation by correcting the reverse ordering possibly appearing in sample group means. The GBS with this correction will be referred to as the constrained group-based strategy (CGBS). If this correction is incorporated in the expectation-maximization (EM) algorithm, it will be called the constrained EM method (CEM).

The following assumptions were made for the purposes of our study: a normal exposure distribution (a log-transformation for log-normally distributed exposures was applied in the example), known constant error variance components (sensitivity study), no systematic error, non-differential measurement error and no correlation among errors. We also focus on the scenario in which the disease under study is neither common nor extremely rare. Throughout this paper, we define exposures as intensity or concentration of substance, ignoring complications that arise from time-varying exposure patterns and accumulation of dose due to long-term exposure. The mechanism for missingness in exposure is ignorable.

Our aim is to examine the use of the constrained method when group means are ordered and the exposures are mis-measured and not available completely in an epidemiological cohort. We discuss only simple linear and logistic response models in this manuscript, but they can be extended to models with several additional variables with no measurement error. We consider two cases for exposure: (1) observations for exposures are completely available but measured with errors and (2) a large portion of exposure data are missing while the other are measured with errors. Also, we assume that the distributions of groups are severely overlapped, that is, the distance among the population group means is small.

## Methods

In this section, we provide models, a brief review for the GBS when the number of observed exposures is large and the group means are far apart, isotonic regression, and the implication of using the isotonic regression. For the considerations described in Background, we attempt to improve the existing methods by introducing CGBS in the specific case of the GBS and CEM that incorporate the group ordering information.

### Models

We assume that the true exposure for the *g*th group is normally distributed with fixed population group mean *μ*_*g*_, *g *= 1,2, ··· ,*G*, and common variance *σ*^2^. These mean exposures are assumed to be ordered according to groups, *i.e., μ*_1_ ≤ *μ*_2_ ≤ ··· ≤ *μ*_*G*_, which is reasonable if the subjects in the higher indexed group experience more exposure on the average. Given this ordering information, we postulate a classical exposure measurement error model as follows:

(1) $$\;W_{\text{gi}}=\mu_{g}+\gamma_{\text{gi}}+\eta_{\text{gi}}=X_{\text{gi}}+\eta_{\text{gi}},$$

where *W*_*gi *_represents the observed exposure on the *i*th subject in the *g*th group, *X*_*gi*_ represents the true exposure on the subject; *μ*_*g*_ is the *g*th group mean exposure, *g* = 1, ··· ,*G*; $\gamma_{\text{gi}}∼N(0,\sigma_{b}^{2})$ is a random effect due to subject *i*, *i* = 1, ··· , *n*_*g*_ in group g; $\eta_{\text{gi}}∼N(0,\sigma_{\eta }^{2})$ is a measurement error occurring when evaluating the amount of exposure. We assume *η*_*gi*_ are mutually independent. Also, *X*_*gi *_and *η*_*gi*_ are assumed to be independent as usual. For the association between exposure and response, we consider simple linear and logistic regression models given, respectively, by

$$\;Y_{\text{gi}}=\beta_{0}+\beta_{1}X_{\text{gi}}+\varepsilon_{\text{gi}},$$

where *β*_0_ and *β*_1_ are the intercept and the slope parameters and $\varepsilon_{\text{gi}}∼N(0,\sigma_{\varepsilon }^{2})$, and

$$\;P[Y_{\text{gi}}=1|X_{\text{gi}}]\equiv p(X_{\text{gi}};\beta )=\wedge (\beta_{0}+\beta_{1}X_{\text{gi}}),$$

where *Y*_*gi*_ is a binary variable for the health outcome and *∧*(*t*) = exp(−*t*)/[1 + exp(−*t*)].

### GBS

As mentioned earlier, GBS assigns the same amount of exposure (sample mean) to all the subjects in the same group. Let ${\bar{W}}_{g}$ be the average of the observed measurements for group *g*. Then, the conditional expectation of the true exposure given the observed group mean in this case is $E[X_{\text{gi}}|{\bar{W}}_{g}]={\bar{W}}_{g}+(m-1)({\bar{W}}_{g}-\mu_{g}),$ where $m=\mathrm{cov}(X_{\text{gi}},{\bar{W}}_{g})/\mathrm{var}({\bar{W}}_{g})$. If the number of subjects in each group is sufficiently large, then ${\bar{W}}_{g}\approx \mu_{g}$ and so $E[X_{\text{gi}}|{\bar{W}}_{g}]$ can be well approximated by ${\bar{W}}_{g}$. Thus, in this case, a *quasi-Berkson* error model [5] can be postulated between the assigned sample mean (${\bar{W}}_{g}$) and true exposures (*X*_*gi*_), i.e.

(2) $$\;X_{\text{gi}}={\bar{W}}_{g}+e_{\text{gi}},\;\;\;\;\;\;E[e_{\text{gi}}|{\bar{W}}_{g}]=0.$$

While the performance of this approximation depends on the sample size, it works well even with a moderate size of sample. This postulation is analogous to the Berkson error model in the sense that the conditional expectation of the true exposure given the observed exposure is exactly the same as the observed exposure. However, it is not a true Berkson error model because the group mean (${\bar{W}}_{g}$) is not independent of the error (*e*_*gi*_). Further, the conditional expectation of response *Y*_*gi*_ given the observed sample mean value, ${\bar{W}}_{g}$ for the linear models is

$$\;E[Y_{\text{gi}}|{\bar{W}}_{g}]=\beta_{0}+\beta_{1}E[X_{\text{gi}}|{\bar{W}}_{g}]$$

and for logistic regression models, by using the approximation to probit regression model when the disease is not rare,

$$\;\begin{array}{ll} E[P(Y_{\text{gi}}\;=\;1|X_{\text{gi}})|{\bar{W}}_{g}]\approx \wedge \left(\beta_{0}^{\prime }+\beta_{1}^{\prime }E(X_{\text{gi}}|{\bar{W}}_{g})\right)\\ \;\;\approx E\left[\Phi [c(\beta_{0}+\beta_{1}X_{\text{gi}})]|{\bar{W}}_{g}\right]\; & \; \end{array}$$

where *c*, the correcting factor for the approximation between logistic and probit models, $\beta_{0}^{\prime }$ and $\beta_{1}^{\prime }$ are functions of the conditional mean $E(X_{\text{gi}}|{\bar{W}}_{g})$ and variance $V(X_{\text{gi}}|{\bar{W}}_{g})$, especially $\beta_{1}^{\prime }=\frac{\beta_{1}}{\sqrt{c^{2}\beta_{1}^{2}V(X_{\text{gi}}|{\bar{W}}_{g})+1}}$, and Φ(*t*) is the cumulative density function of the standard normal distribution [6]. In conclusion, if the number of observed values is large, $E[X_{\text{gi}}|{\bar{W}}_{g}]\approx {\bar{W}}_{g}$, and when $V(X_{\text{gi}}|{\bar{W}}_{g})\approx \sigma_{b}^{2}$ is small, then there is no bias for linear and negligible bias for logistic models [5]. However, as the number of observed values gets smaller, the approximation by using the GBS results in more severe bias. Our goal is to search for an improved method by incorporating the group ordering information.

### Constrained GBS

The isotonic regression is the weighted least-squares fit of unconstrained mean estimates subject to a set of specified ordering constraints. In case of a simple linear order considered here, the isotonic regression of $\bar{w}$ with weight vector ***α*** = (*α*_1_, ··· ,*α*_*G*_)^*′*^ is defined as the solution to

$$\;{\text{Min}}_{\mu }\overset{G}{\underset{g=1}{\sum }}{\left({\bar{w}}_{g}-\mu_{g}\right)}^{2}\alpha_{g}\;\text{subject to}\;\mu_{1}\leq \mu_{2}\leq \cdot \cdot \cdot \leq \mu_{G}.$$

For computing this isotonic regression, let

$$\;\text{Av}\left(i,j\right)=\frac{\alpha_{i}{\bar{w}}_{i}+\alpha_{i+1}{\bar{w}}_{i+1}+\cdot \cdot \cdot +\alpha_{j}{\bar{w}}_{j}}{\alpha_{i}+\alpha_{i+1}\cdot \cdot \cdot +\alpha_{j}},\;1\leq i\leq j\leq G.$$

Then, as discussed in Robertson et. al. [7], the *g*th component value of the isotonic regression is represented as

(3) $$\;\mu_{g}^{*}=\underset{l\leq g}{\text{max}}\underset{j\geq g}{\text{min}}\text{Av}(l,j),\;g=1,\cdot \cdot \cdot \;,G,$$

and can be easily obtained by the Pool-Adjacent-Violators Algorithm (PAVA) (see Chapter 2 of [4] for details).

Table 1 shows an example. Let us assume that the population means of five groups should be increasingly ordered. But we observed reverse orderings between the first two. With weight vector *α* = (4, 3, 5, 3, 4), the components (*μ*^∗^) of group means ($\bar{w}$) are corrected by isotonization. In case of GBS, the observed group means may not be properly ordered if the sample sizes for exposures are small. The reverse ordering in group mean estimates could result in serious bias to the estimators of regression parameters. This type of bias could be avoided by using the isotonic regression that satisfies the ordering constraint for the population means. In the constrained GBS, we adjust the estimated group means to satisfy the required ordering before the GBS is applied. In the model setting here, the component values in the isotonic regression will be used as exposure scores. By adding the ordering information underlying exposure group means in the estimation procedure, improvement of GBS could be possible to a certain extent without any complicated modification even when we have a small number of observations for the exposure in each group. This constrained approach is referred to as CGBS. In CGBS, the mean vector, μ = (*μ*_1_, ··· ,*μ*_*G*_)^*′*^, is estimated by the isotonic regression, μ^∗^ , of $\bar{w}={\left({\bar{w}}_{1},{\bar{w}}_{2},\cdot \cdot \cdot \;,{\bar{w}}_{G}\right)}^{\prime }$ with a weight vector composed of observed sample sizes.

**Table 1:** An example of isotonic regression

**Group**	**1**	**2**	**3**	**4**	**5**
$\bar{w}$	0.7755	0.6229	1.8207	2.2878	1.7054
***α***	4.0	3.0	5.0	3.0	4.0
***μ***^***∗***^	0.7101	0.7101	1.8207	1.9550	1.9550

### Constrained Expectation and Maximization (CEM)

An EM method, introduced by Dempster et al. [8], is used for finding MLEs of parameters where the missing or latent variables are involved in the model. The EM algorithm is an iterative procedure that performs an expectation (E) step and maximization (M) step, alternately. In E-step, the conditional expectation of complete log-likelihood is computed based on the conditional distribution of unobservable variables given observed data and current estimates of parameters. In M-step, the expected likelihood function obtained in E-step is maximized and the current estimates of parameters are updated by the new ones. As discussed in Wu [9], if the likelihood function is unimodal and satisfies some differentiability conditions, EM algorithm produces a sequence of estimates that converge to the actual MLE.

For constrained estimation (CEM), we impose the ordering constraints on *μ*_*i *_’s in this M-step. While we can get closed form of constrained estimates in M-step in the simple linear case with measurement error but without missing in exposure, we usually need another iterative procedure for the maximization in M-step. A simple approach for the maximization in an M-step in our problem is to update estimates alternately between *μ *and other set of parameters. In other words, we first find the constrained estimate of *μ *given current estimates of other parameters, and then using the updated estimate of *μ*, we maximize the expected log-likelihood with respect to other parameters in a usual manner. Here, we briefly discuss how to find the constrained estimate of *μ *and other parameters in our iterative procedure in M-step.

#### CEM with measurement errors only in exposure

The EM method can be applied to measurement error problems by treating unobservable true *x*_*gi*_ as missing data values [2]. If there is no information on measurement error, a sensitivity analysis may be conducted for some predetermined values of error variances. We anticipate that the measurement error and the true variable distributions might be specified as normal linear models. The observed data likelihood is obtained by integrating the completed data likelihood over all the unobserved *x*’s, and then maximized for MLE. The complete data likelihood for *θ* = (*θ*_1_, *θ*_2_, *θ*_3_) is

(4) $$\;L_{c}(\theta |y,w,x)=\overset{G}{\underset{g=1}{\prod }}\overset{n_{g}}{\underset{i=1}{\prod }}f(y_{\text{gi}}|x_{\text{gi}};\theta_{1})f(w_{\text{gi}}|x_{\text{gi}};\theta_{2})f(x_{\text{gi}};\theta_{3}).$$

While we use *f*(·|·) for conditional densities, they can be identified based on their arguments. Integrating over the cases with unobserved *x*’s will give the observed data likelihood:

$$\begin{array}{c} \;L_{o}\left(\theta |y,w,x\right)\;=\;\overset{G}{\underset{g=1}{\prod }}\;\;\overset{n_{g}}{\underset{i=1}{\prod }}\;\int \;\;f\;\left(y_{\text{gi}}|x_{\text{gi}};\theta_{1}\right)f\;\left(w_{\text{gi}}|x_{\text{gi}};\theta_{2}\right)f\;\left(x_{\text{gi}};\theta_{3}\right)dx_{\text{gi}} \end{array}$$

Let *l*_*c*_(*θ*; **y**, **w**, **x**) = ln *L*_*c*_(*θ*; **y**, **w**, **x**) − *C* where *C* is the constant term in log*L*_*c*_(*θ*; **y**, **w**, **x**). The E-step involves formulation of the quantity *Q*(*θ*|*θ*^(*t*)^) such that

(5) $$\;Q(\theta |\theta^{\left(t\right)})=E_{\theta^{\left(t\right)}}[l_{c}(\theta ;Y,W,X)|y,w]$$

where the expectation is with respect to the conditional density *f*(*x*_*gi*_|*w*_*gi*_,*y*_*gi*_;*θ*^(*t*)^). The conditional pdf of *X*_*gi *_given *y*_*gi *_and *w*_*gi*_ is given by

$$\;f\left(x_{\text{gi}}|y_{\text{gi}},w_{\text{gi}};\theta \right)=\frac{f(y_{\text{gi}}|x_{\text{gi}};\theta_{1})f(w_{\text{gi}}|x_{\text{gi}};\theta_{2})f(x_{\text{gi}};\theta_{3})}{\int f(y_{\text{gi}}|x_{\text{gi}};\theta_{1})f(w_{\text{gi}}|x_{\text{gi}};\theta_{2})f(x_{\text{gi}};\theta_{3})dx_{\text{gi}}}.$$

When we maximize *Q*(*θ*|*θ*^(*t*)^), we may often encounter the problem of evaluating expectations that do not have a closed form. Then, a Monte Carlo method can be used in various ways. Also, in order to avoid the identifiability problem commonly arising from measurement error models, we assume $\sigma_{\eta }^{2}$ is known.

#### CEM with measurement errors and missing in exposure

Methods for data with complex structure such as measurement errors, missingness and outliers were reviewed by Wu [10]. The EM method is used for estimation when measurement error or missingness is involved in responses [11]. Observations containing the cases with missing values in exposure can also be accommodated by the EM algorithm. If there are any constraints on mean exposures, we may use the constrained EM method that reflects such constraints in maximization procedure.

Let $\left\{(Y^{*},W^{*},X^{*});n_{g}^{*}\;\text{number of missing exposures}\right\}$ be a subdata where *Y*^∗^ is observed, but *W*^∗^ is all missing with the true value *X*^∗^ while {(*Y*, *W*, *X*);*n*_*g *_number of observed exposures} denotes those without any missing value. Then, as we defined earlier, the complete data log-likelihood for *θ* is given by

$$\;l_{c}(\theta ;y,w,x,y^{*},w^{*},x^{*})=l_{c_{1}}(\theta ;y,w,x)+l_{c_{2}}(\theta ;y^{*},w^{*},x^{*}).$$

where *l*_*c*_ is the log-likelihood with complete data, $l_{c_{1}}$ is the log-likelihood with observed exposures, and $l_{c_{2}}$ is the log-likelihood with the missing exposures. Thus, it follows that

$$\;\begin{array}{l} Q(\theta |\theta^{\left(t\right)})\;=E_{\theta^{\left(t\right)}}[l_{c}(\theta ;Y,W,X,Y^{*},W^{*},X^{*})|y,w,y^{*}]\\ \;\;=Q_{1}(\theta |\theta^{\left(t\right)})+Q_{2}(\theta |\theta^{\left(t\right)})\; \end{array}$$

where $Q_{1}(\theta |\theta^{\left(t\right)})=E_{\theta^{\left(t\right)}}[l_{c_{1}}(\theta ;Y,W,X)|y,w]$ and $Q_{2}(\theta |\theta^{\left(t\right)})=E_{\theta^{\left(t\right)}}[l_{c_{2}}(\theta ;Y^{*},W^{*},X^{*})|y^{*}]$. Note that $Q_{1}(\theta |\theta^{\left(t\right)})$ is exactly the same as the conditional expectation obtained in E-Step in the case with measurement error only. Based on observations having missing values in exposure, the second term of *Q*(*θ*|*θ*^(*t*)^) for linear regression model is expressed as

(6) $$\;\begin{array}{l} \;Q_{2}(\theta |\theta^{\left(t\right)})=-\frac{1}{2}\left(\overset{G}{\underset{g=1}{\sum }}n_{g}^{*}\right)(\ln\overset{2}{\underset{\eta }{\sigma }}+\ln\overset{2}{\underset{b}{\sigma }}+\ln\overset{2}{\underset{\theta }{\sigma }})\\ \;\;\;-\frac{1}{2\sigma_{\theta }^{2}}\overset{G}{\underset{g=1}{\sum }}\overset{n_{g}^{*}}{\underset{i=1}{\sum }}E_{\theta^{\left(t\right)}}[{\left(Y_{\text{gi}}^{*}-\beta_{0}-\beta_{1}X_{\text{gi}}^{*}\right)}^{2}|y_{\text{gi}}^{*}]\\ \;\;\;-\frac{1}{2\sigma_{\eta }^{2}}\overset{G}{\underset{g=1}{\sum }}\overset{n_{g}^{*}}{\underset{i=1}{\sum }}E_{\theta^{\left(t\right)}}[{\left(W_{\text{gi}}^{*}-X_{\text{gi}}^{*}\right)}^{2}|y_{\text{gi}}^{*}]\\ \;\;\;-\frac{1}{2\sigma_{b}^{2}}\overset{G}{\underset{g=1}{\sum }}\overset{n_{g}^{*}}{\underset{i=1}{\sum }}E_{\theta^{\left(t\right)}}[{\left(X_{\text{gi}}^{*}-\mu_{g}\right)}^{2}|y_{\text{gi}}^{*}].\; \end{array}$$

The conditional distribution (*X*_*gi*_|*y*_*gi*_, *w*_*gi*_) follows N(*m*_*x*_(*y*_*gi*_, *w*_*gi*_;*θ*), *v*_*x*_(*θ*)) where $m_{x}(y_{\text{gi}},w_{\text{gi}};\theta )=\frac{\beta_{1}\sigma_{b}^{2}\sigma_{\eta }^{2}(y_{\text{gi}}-\beta_{0})+(\sigma_{\theta }^{2}\sigma_{\eta }^{2})w_{\text{gi}}+\sigma_{\theta }^{2}\sigma_{\eta }^{2}\mu_{g}}{\beta_{1}^{2}\sigma_{b}^{2}\sigma_{\eta }^{2}+\sigma_{\theta }^{2}(\sigma_{b}^{2}+\sigma_{\eta }^{2})}$ and $v_{x}(\theta )=\frac{\sigma_{\theta }^{2}\sigma_{b}^{2}\sigma_{\eta }^{2}}{\beta_{1}^{2}\sigma_{b}^{2}\sigma_{\eta }^{2}+\sigma_{\theta }^{2}(\sigma_{b}^{2}+\sigma_{\eta }^{2})}$. Also, the conditional distribution $\left(X_{\text{gi}}^{*},W_{\text{gi}}^{*}|y_{\text{gi}}^{*}\right)$ follows a bivariate normal distribution with $m_{w^{*}}(y_{\text{gi}}^{*};\theta ),\rho_{x^{*}w^{*}}(\theta ),v_{x^{*}}(\theta )$, and $v_{w^{*}}(\theta )$, where $m_{x^{*}}(y_{\text{gi}}^{*};\theta )=m_{w^{*}}(y_{\text{gi}}^{*};\theta )=\frac{\sigma_{\theta }^{2}\mu_{g}+\beta_{1}\sigma_{b}^{2}(y_{\text{gi}}^{*}-\beta_{0})}{\sigma_{\theta }^{2}+\beta_{1}^{2}\sigma_{b}^{2}}$, $\rho_{x^{*}w^{*}}(\theta )={\left[\frac{\sigma_{\theta }^{2}\sigma_{b}^{2}}{\sigma_{\theta }^{2}\sigma_{b}^{2}+\sigma_{\theta }^{2}\sigma_{\eta }^{2}+\beta_{1}^{2}\sigma_{b}^{2}\sigma_{\eta }^{2}}\right]}^{\frac{1}{2}}$, $v_{x^{*}}(\theta )=\frac{\sigma_{\theta }^{2}\sigma_{b}^{2}}{\sigma_{\theta }^{2}+\beta_{1}^{2}\sigma_{b}^{2}}$, and $v_{w^{*}}(\theta )=\frac{\sigma_{\theta }^{2}\sigma_{b}^{2}+\sigma_{\theta }^{2}\sigma_{\eta }^{2}+\beta_{1}^{2}\sigma_{b}^{2}\sigma_{\eta }^{2}}{\sigma_{\theta }^{2}+\beta_{1}^{2}\sigma_{b}^{2}}$.

The second term $Q_{2}(\theta |\theta^{\left(t\right)})$ for logistic regression model is given by

(7) $$\;\begin{array}{l} Q_{2}(\theta |\theta^{\left(t\right)})=-\frac{1}{2}\left(\overset{G}{\underset{g=1}{\sum }}\right)n_{g}^{*}(\ln\overset{2}{\underset{\eta }{\sigma }}+\ln\overset{2}{\underset{b}{\sigma }})\\ \;\;\;+\overset{G}{\underset{g=1}{\sum }}\overset{n_{g}^{*}}{\underset{i=1}{\sum }}\left\{y_{\text{gi}}^{*}E_{\theta^{\left(t\right)}}[\ln p(\overset{*}{\underset{\text{gi}}{X}};\beta )|y_{\text{gi}}^{*}]\right)\\ \;\;\;\left(+(1-y_{\text{gi}}^{*})E_{\theta^{\left(t\right)}}[\ln(1-p(\overset{*}{\underset{\text{gi}}{X}};\beta ))|y_{\text{gi}}^{*}],\;\right\}\\ \;\;\;-\frac{1}{2\sigma_{\eta }^{2}}\overset{G}{\underset{g=1}{\sum }}\overset{n_{g}^{*}}{\underset{i=1}{\sum }}E_{\theta^{\left(t\right)}}\left[{\left(W_{\text{gi}}^{*}-X_{\text{gi}}^{*}\right)}^{2}|y_{\text{gi}}^{*}\right]\\ \;\;\;-\frac{1}{2\sigma_{b}^{2}}\overset{G}{\underset{g=1}{\sum }}\overset{n_{g}^{*}}{\underset{i=1}{\sum }}E_{\theta^{\left(t\right)}}\left[{\left(X_{\text{gi}}^{*}-\mu_{g}\right)}^{2}|y_{\text{gi}}^{*}\right]. \end{array}$$

In order to maximize *Q*(*θ*|*θ*^(*t*)^), we need a Newton method as a part of each EM procedure. However, our investigations indicate that it does not take too long time to reach a convergence criterion. As mentioned earlier, the conditional expectations here do not have closed form of expressions, and thus we rely on a Monte Carlo method to evaluate them. For details of estimation procedure, readers are referred to the Additional file 1: Supplementary material of this paper.

## Simulation study and results

Simulations were conducted to examine attenuation in regression coefficient estimates in linear and logistic models with group-based exposure assessment and a disease with expected risk of about 10%. We considered a cohort with time-invariant exposure that segregates into five exposure groups. We further assumed that disease risk depended only on exposure intensity, not its duration. For each group, we generated *n*_*y *_= 30 sets of complete observations (exposure with measurement error and response) from the specified underlying model described in Section “Methods”. In order to allow missingness for exposure, a sample of *n*_*x *_= 10 or 20 exposure measurements were taken from the complete set of data (*n*_*y *_= 30) for each group. Here, the true regression coefficients were set to *β*_0 _= − 2 and *β*_1 _= 0.3 as the intercept and slope parameters for both regression models. For both models, the exposures were assumed to be normally distributed with means *μ*_*g *_= 0.2 + (*g* − 1)*δ*, *g *= 1,···,5. We consider two cases: *δ *= 0.3 and 0.5. In order to see the impact of the between-subject variability, we investigated the results for different values of $\sigma_{b}^{2}=0.3,\;0.6,\;\text{and}\;0.9$. Three levels of measurement error variance were considered, that is, $\sigma_{\eta }^{2}=$ 0.25, 0.5, and 0.75. The empirical bias and mean square error (MSE) were evaluated based on 200 replications for each case.

We perform four estimation methods. The first method, named ‘naive’, is to analyze the complete data ignoring exposure measurement errors. The second approach (CEM) is the constrained EM algorithm developed for accommodating missing observations and measurement errors. The third method is to use the GBS and CGBS methods that assign a score (group mean for GBS or constrained group mean for CGBS) to each group and fit a standard regression model to the data. In implementing the Monte Carlo CEM methods, we used the naive estimates as initial values for parameters. We present the empirical bias and MSE in (Additional file 2: Tables S1 and S2) for linear model and in (Additional file 2: Tables S3 and S4) for logistic model, respectively. Evidently, the results demonstrate that substantial bias is incurred if missingness and measurement error are not properly treated. The CEM adjusted fairly well the bias if there is no missing. The CGBS is better than the GBS overall. When the portion of observed exposure data is small and they are exposed to severe measurement error, the CGBS performs much better than the other methods.

Specifically, in the linear model case (Additional file 2: Table S1), when the between-group contrast is small (*δ* = 0.3), the naive estimate is seriously biased, but CEM method (with full measurements) has shown significant improvement. For example, the between-subject variability and the measurement error are moderate ($\sigma_{b}^{2}=0.6$; $\sigma_{\eta }^{2}=0.5$), the CEM estimate adjusts bias (0.9%) whereas the bias of naive estimates is about 40%. However, when the number of observed exposure data is *n*_*x *_= 10, in the same comparison, the bias of CGBS estimate is smaller than that of CEM estimate (2.8% vs. 5.1%). In the same setting, when measurement error is increased to $\sigma_{\eta }^{2}=0.75$, all the estimators get more biased. But, the CGBS performs better than the others (3.0% vs. 20.4%(GBS) and 7.9%(CEM)). If the between group contrast is increased, the estimators get less biased for all methods but the pattern of bias among the estimation methods is similar to that of the case with the small between-group contrast. As the between-subject variability increases, the bias of GBS and CGBS estimates increases, whereas the bias of CEM estimate decreases. For example, in (Additional file 2: Table S2), with *δ *= 0.5 and *n*_*x *_= 10 measurements, and the measurement error are moderate ($\sigma_{\eta }^{2}=0.5$), as the between-subject variability increases from $\sigma_{b}^{2}=0.3$ to 0.9, the bias of CGBS estimate increased from 2.0*%* to 2.7*%*, whereas the CEM estimate decreased from 5.4*%* to 3.7*%*.

In the logistic regression (Additional file 2: Table S3), when the between-group contrast is small (*δ* = 0.3), the naive estimate is highly biased, whereas CEM method (with full measurements) adjusts the bias significantly. For example, the between-subject variability and the measurement error are moderate ($\sigma_{b}^{2}=0.6$; $\sigma_{\eta }^{2}=0.5$), the CEM estimate adjusts bias (0.5%), whereas the bias of naive estimates is about 44%. However, when the number of observed exposure data is *n*_*x *_= 10, in the same comparison, the bias of CGBS estimate is much smaller than that of CEM estimate (5.1% vs. 23.1%). In the same setting, If we increase measurement error to $\sigma_{\eta }^{2}=0.75$, all the estimates get worse. However, the CGBS shows better performance compared with the other methods (5.7% vs. 25%(GBS) and 27%(CEM)). As the between-group contrast increases (Additional file 2: Table S4), the estimates are generally getting less biased. For example, when *n*_*x *_= 20, $\sigma_{b}^{2}=0.6$, and $\sigma_{\eta }^{2}=0.5$, the bias of CEM estimate with *δ *= 0.5 is 5.5*%* whereas the bias when *δ *= 0.3 is 7.3%. In the same comparison, the bias of CGBS estimate when *δ *= 0.5 is 11.8% whereas its bias with *δ *= 0.3 is 26%.

For both linear and logistic models, the MSE decreases as we increase the between-group contrast. The MSEs for the CEM estimators were generally smaller than those for the GBS and CGBS when the number of observed exposure is not too small. However, with small number of observations, the CGBS performs better than the CEM. Also, MSE gets larger if we increase the measurement error variability.

## Application to respiratory symptoms and exposure to carbon black

A number of repeated cross-sectional studies with measurements of respiratory health of employees in the European carbon black manufacturing industry, in relation to exposure to carbon black dust, were conducted [12]. In the second survey, exposure to respiratory dust in 19 factories in 7 European countries (Great Britain, France, Germany, Holland, Italy, Spain and Sweden) was determined among 1870 workers, resulting in 3290 measurements [13]. There were 8 job categories within 19 factories, and workers from each job title in each factory were selected randomly for monitoring of exposures. In addition, repeated exposure measurements on the same worker were collected at random intervals to allow estimation of the between- and within- worker variances. Respiratory dust measurements were log- transformed to satisfy the assumption of normality. Lung function tests were carried out by the company medical officers, or their delegates, with a properly maintained Vitalograph S Model dry wedge bellows spirometer calibrated with a 1 l syringe. The spirometer was calibrated by external specialists at the start of each data collection period and at 2 week intervals throughout the surveys. Values for forced expiratory volume in 1 second (*FEV*_1_) among others were estimated directly from the traces.

Exposure models were fitted with both homogenous and heterogeneous between-subject variances among groups to check the assumption of the homogenous between-subject variability for all groupings. Comparison with Bayesian information criterion values in mixed-effect modelling indicated that there was no evidence for heterogeneous between-subject variability in the data [14]. We used a subset (1414 workers) of the original data in which each subject within a group had exposure measurement and health outcomes were determined on every subject with one exposure measurement.

**Figure 1:**
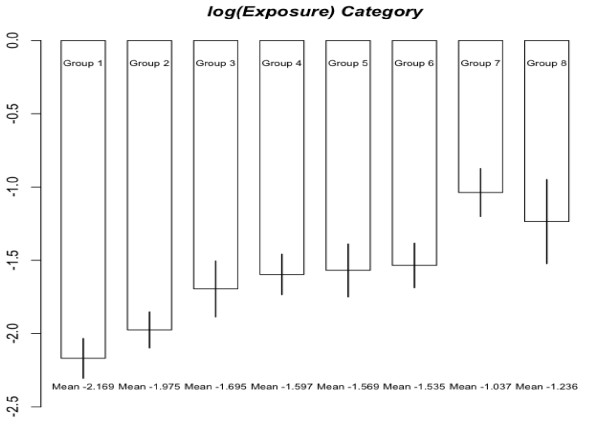
Mean of log-exposures to respiratory dust by exposure category.

Figure 1 shows the means of log-exposures are ordered among the groups defined by job categories while there is an exception. In the presence of this ordering information, estimates of the slope parameter were obtained for different numbers of exposure data, *n*_*x *_= 10,20,50, selected randomly from each group of data. These estimates are listed in Table 2 to demonstrate the effect of the number of incomplete observations on the performance of analytical methods. For this, the mean estimates and their standard errors were evaluated from 200 randomly selected samples of size *n*_*x*_. However, in the case of no missing observations, the standard error of ${\hat{\beta }}_{1}$ was computed based on the empirical information matrix in the context of Meilijson [15]. In the case of GBS or CGBS, the variance estimate for the response depends not only on model and measurement error variances but also on the variability of exposure and the slope of the regression function. This is because such methods treat responses in the same group as if they were observed at the group mean exposure. Thus, the standard errors from those methods are inflated. Thus, we suggest to use the standard errors from CEM for general inference.

**Table 2:** Application to respiratory symptoms and exposure to carbon black

**G = 8, n**_**y**_** = (245, 240, 129, 224, 157, 192, 174, 53), δ = 0.36**						
	$s_{b}^{2}$	$s_{\eta }^{2}$	**Naive(se)**	**GBS**(**se**^**∗**^)	**CGBS**(**se**^**∗**^)	**CEM(se)**
*n*_*x *_= full	0.41	0.92	-0.0408(0.019)	-0.1593(0.059)	-0.1709(0.059)	-0.1589(0.014)
					^∗ ^inflated standard error due to GBS approach	
				**GBS**	**CGBS**	**CEM(se)**
*n*_*x *_= 50				-0.1501	-0.1725	-0.1456(0.023)
*n*_*x *_= 20				-0.1270	-0.1637	-0.1371(0.025)
*n*_*x *_= 10				-0.1004	-0.1600	-0.1338(0.025)

Grouping with job category: Estimate of ***β***_**1 **_(with standard error in parentheses) *G*: number of groups; ***n***_***x***_: number of exposures per group; ***s***: estimate variance of the between group distribution of log-transformed exposures; $s_{b}^{2}$: estimate variance of the between worker distribution of log-transformed exposures; GBS: group-based strategy; CGBS: constrained group-based; Monte Carlo EM (CEM).

Furthermore, our simulation study indicates that the estimation by CEM is more reliable if data do not contain missing observations. Thus, we suggest in this example to use ${\hat{\beta }}_{1}=-0.1589$ as the best estimate of *β*_1_. Although GBS gave the estimated value incidentally closer to the suggested estimate than CGBS did, CGBS estimates can be expected to be more precise in general as shown in our simulation study. This argument becomes more clear when we compare estimates obtained from data sets containing missing observations. We see that CGBS estimates are stably closer to the suggested estimate regardless of the level of missingness. More specifically, with small sample size, say *n*_*x *_= 10, GBS yielded estimate that is closer to naive analysis with little adjustment for measurement error compared with CGBS and CEM. This can be anticipated because GBS retains predominantly classical error structure in such case. Recall CEM estimators are more precise than CGBS estimators in simulations if there are no missing observations but less precise then CGBS estimators if a substantial portion of observations is missing in each group. The results also support the forementioned fact in the sense that those CGBS estimates are stably closer to the suggested “best” estimate in all cases with missing observations. Since the data set used here is the case of small intervals ($\hat{\delta }=0.36$) and large measurement errors ($\sigma_{\eta }^{2}=0.92$), this interpretation may apply limitedly to that particular situation.

## Discussion

Measurement error is concerned with bias problem occurring to the estimates of regression coefficients when the exposures are measured inaccurately or available through the recording of imperfect surrogates. If we, in addition to measurement error for observed exposure, have missing values for many exposure measurements possibly due to excessive cost or lack of data in retrospective analyses, data analyses themselves face challenges that can not be overcome by standard approaches.

Natural orderings are found in many practical application. In a bioassay problem illustrated in Robertson et. al. [7], a pharmaceutical researcher may want to test if treatment effects are ordered according to dosage levels. In our example, jobs are categorized as administration area worker, laboratory assistant/electrician, fitter/welder, process/conveyor operator, and warehouse packer/shipping or cleaning (not office). The population means of carbon black exposure among these job categories could be ordered increasingly (from administration in office to cleaning outside). Although there is one exception possibly due to randomness, this ordering trend is justified by exposure data as shown in Figure 1. If this ordering information is ignored, the analysis would result in serious bias. We demonstrated that incorporating ordering information on group means in estimation procedure brought a significant improvement. Although we focused on linear and logistic regression models, it would be possible to extend this argument to more general models.

In environmental epidemiology, when we cannot directly measure the exposure where there is outcome data, various methods are used to predict the unknown exposure that are similar to group-based approach employed in occupational epidemiology.

When land-use regression models or spatial patterns of exposure are used to assign exposure to all residents of an area, measurement error can have features of both classical-like and Berskon-like errors as discussed in Szpiro et al. [16,17]. The Berkson-like component of error arises because the exposure model does not account for all sources of variation in true exposure. This is similar to our work in that the quasi-Berkson error we identified from the re-postulated model (2) is approximately independent of the assigned exposures [5]. It must be recalled, however, that quasi-Berkson error can be assured only when group means are estimated precisely from a large sample. The contribution of our current work is to consider a challenging situation where we are dealing with a small number of observed exposure measurements and between-group contrast is small and the quasi-Berkson model cannot be reliable. The analogy to work of Szpiro et al. [16] may be the case where empirical exposure model is constructed on limited data, as may well be commonly the situation in environmental epidemiology. In our case, however, there is no auxiliary variable to be used in modeling the true exposure but instead we have the ordering information among group mean exposures. Thus, the exposure problem is more related to that of estimation rather than prediction. As a result, the expressions for Berkson-like and classical-like error components in Szpiro et al. [16] have different interpretations in our problem. In fact, it is decomposed into within-group variability for the true exposure and estimation error for mean exposure. This is caused by the fact we used ${\bar{W}}_{g}$ instead of *W*_*gi *_’s. It should be noted that reducing estimation error by incorporating the information on ordering of groups contributes to reduction in the bias of the estimated regression parameter arising from measurement errors and small sample samples of exposures from highly overlapped groups.

## Conclusion

The constrained group-based (CGBS) is one of methods that can accommodate the group ordering property. This method assigns the ordered group means to exposure values instead of the naive group means. The simulations show that estimates with the CGBS method are improved significantly when the between-group contrast is small, measurement error variance is large and there are many missing observations. However, it should be used carefully because the bias reduction was achieved with some increased uncertainty (variance). The constrained expectation-maximization (CEM) method appears to be best when all or moderate number of exposures are observed with measurement errors. On the other hands, the CGBS has an advantage that it is easy to implement with no serious loss compared with the CEM that requires highly intensive computation. In fact, CGBS appears to have more desirable bias-reducing properties in presence of substantial proportion of missing values.

By incorporating group ordering information in GBS or EM, we can conduct more precise analysis for data sets that could be considered, otherwise, as too small a sample. For example, thousands of measurements were collected in the the analysis considered in the example we discussed here. But, the analysis could have been possible even with much less measurement efforts, say, as few as 10 measurements per exposure group (a total of 80 measurements), with CGBS or CEM. If we are interested only in bias reduction, this fact has an important implication for cost-effective studies when groups are ordered in terms of exposure mean. However, as we mentioned earlier, standard errors of GBS or CGBS estimates are inflated and it is recommended to use those from the CEM method.

In conclusion, if groups are ordered according to exposure level, a constrained group- based exposure assignment can be an effective and versatile approach to estimate the relationship between exposure and disease when exposure data are not available for all subjects under study. Although a large sample is typically required for precise estimation, we showed that even smaller samples could be used to obtain informative results in realistic settings by incorporating order relation among group exposure means. Note the availability of the ordering information contributed to reducing bias and increasing precision. However, if such partial information is not available or irrelevant to the response due to the nature of the problem, this improvement cannot be expected. It should also be noted that if the number of observed measurements are not too small, the constrained EM approach may be recommended as a general analytical approach to semi-ecological study design. Finally, as a future study, we may develop an efficient simulation procedure that can analyze comprehensively the impacts of relative variability in the group means, subject-specific exposure variation, and measurement error variance. Furthermore, we may extend the proposed approach so that it can be applicable to more complex data containing repeated measurements and clusters.

## Competing interests

The authors declare that they have no competing interests.

## Author’s contributions

HMK conceived the study, conducted the simulation study, carried out the statistical analysis, and drafted the manuscript. CGP contributed to the development of the statistical methods and interpretation of the findings, and participated in the drafting of the manuscript. MVT contributed to the drafting of the manuscript and interpretation of re-analysis of epidemiological data. IB helped develop the research question, participated in the interpretation of epidemiological examples and drafting of the manuscript. All authors read and approved the final manuscript.

## Pre-publication history

The pre-publication history for this paper can be accessed here:

http://www.biomedcentral.com/1471-2288/12/135/prepub

## Supplementary Material

Additional file 1 **Supplementary material. **Detail description for EM algorithm. Click here for file

Additional file 2 **Tables from Simulation Studies. Table S1. **Empirical bias of ${\hat{\beta }}_{1}$ (with empirical **MSE **in parentheses) in the linear model when between-group contrast is 0.3. Table S2. Empirical bias of ${\hat{\beta }}_{1}$ (with empirical **MSE **in parentheses) in the linear model when between-group contrast is 0.5. Table S3. Empirical bias of ${\hat{\beta }}_{1}$ (with empirical **MSE **in parentheses) in the logistic model when between-group contrast is 0.3. Table S4. Empirical bias of ${\hat{\beta }}_{1}$ (with empirical **MSE **in parentheses) in the logistic model when between-group contrast is 0.5. Click here for file
