# Supplementary material for: Estimation methods with ordered exposure subject to measurement error and missingness in semi-ecological design
Source: BMC Med Res Methodol. 2012 Sep 4;12:135. doi: 10.1186/1471-2288-12-135 (PMC3552934; doi:10.1186/1471-2288-12-135)
Supplement: Additional file 1 — Supplementary material. Detail description for EM algorithm. [file 1471-2288-12-135-S1.pdf]

Estimation Methods with Ordered Covariate Subject to Measurement Error and Missingness in Semi-Ecological Design

by

Hyang-Mi KIM[1*] Chul Gyu PARK[2] Martie van TONGEREN[3] Igor BURSTYN[4]

Supplementary Material

EM with Measurement Errors Only:

1) Linear regression

First note that $\theta = (\theta_1, \theta_2, \theta_3)$ where $\theta_1 = (\beta_0, \beta_1, \sigma_\varepsilon^2)$, $\theta_2 = \sigma_\eta^2$ and $\theta_3 = (\mu, \sigma_b^2)$. The E-Step of the tth iteration of the EM procedure gives

$$\begin{aligned} Q(\theta|\theta^{(t)}) &= E_{\theta^{(t)}}[l_c(\theta;\mathbf{Y},\mathbf{W},\mathbf{X})|\mathbf{y},\mathbf{w}] \\ &= -\frac{1}{2}\left(\sum_{g=1}^{G} n_g\right)(\ln\sigma_\eta^2 + \ln\sigma_b^2 + \ln\sigma_\varepsilon^2) - \frac{1}{2\sigma_\varepsilon^2}\sum_{g=1}^{G}\sum_{i=1}^{n_g} E_{\theta^{(t)}}[(Y_{gi}-\beta_0-\beta_1 X_{gi})^2|y_{gi},w_{gi}] \\ &- \frac{1}{2\sigma_\eta^2}\sum_{g=1}^{G}\sum_{i=1}^{n_g} E_{\theta^{(t)}}[(W_{gi}-X_{gi})^2|y_{gi},w_{gi}] - \frac{1}{2\sigma_b^2}\sum_{g=1}^{G}\sum_{i=1}^{n_g} E_{\theta^{(t)}}[(X_{gi}-\mu_g)^2|y_{gi},w_{gi}] \qquad (1) \end{aligned}$$

In M-Step, we need to maximize $Q(\theta|\theta^{(t)})$ under the constraints $\mu_1 \le \mu_2 \le \cdots \le \mu_G$. For this, note first the conditional variable $(X_{gi}|y_{gi}, w_{gi})$ follows $N(m_x(y_{gi}, w_{gi};\theta), v_x(\theta))$, where

$$m_x(y_{gi}, w_{gi};\theta) = \frac{\beta_1\sigma_b^2\sigma_\eta^2(y_{gi}-\beta_0) + (\sigma_\varepsilon^2\sigma_\eta^2)w_{gi} + \sigma_\varepsilon^2\sigma_\eta^2\mu_g}{\beta_1^2\sigma_b^2\sigma_\eta^2 + \sigma_\varepsilon^2(\sigma_b^2+\sigma_\eta^2)} \quad \text{and} \quad v_x(\theta) = \frac{\sigma_\varepsilon^2\sigma_b^2\sigma_\eta^2}{\beta_1^2\sigma_b^2\sigma_\eta^2 + \sigma_\varepsilon^2(\sigma_b^2+\sigma_\eta^2)}.$$

Let $\bar{m}_g = \frac{1}{n_g}\sum_{i=1}^{n_g} m_x(y_{gi}, w_{gi};\theta^{(t)}), g = 1, \cdots, G$, $\bar{m} = \frac{1}{\sum_{g=1}^{G} n_g}\sum_{g=1}^{G} n_g\bar{m}_g$, and $\bar{y} = \frac{1}{\sum_{g=1}^{G} n_g}\sum_{g=1}^{G}\sum_{i=1}^{n_g} y_{gi}$. Then, the solution to this maximization problem can be found and updated as follows:

$$\mu^{(t+1)} = \text{isotonic regression of } (\bar{m}_1, \bar{m}_2, \cdots, \bar{m}_G)' \text{ with weight vector } (n_1, n_2, \cdots, n_G)',$$

[*]Corresponding author: hmkim@ucalgary.ca, Office: (403) 220-5691, Fax: (403) 282-5150: Department of Mathematics and Statistics, The University of Calgary, 2500 University Drive N.W. Calgary, Alberta, Canada T2N 1N4

$\beta_1^{(t+1)} = \frac{\sum_{g=1}^{G}\sum_{i=1}^{n_g}[m_x(y_{gi},w_{gi};\theta^{(t)})-\bar{m}]y_{gi}}{\sum_{g=1}^{G}\sum_{i=1}^{n_g}[m_x(y_{gi},w_{gi};\theta^{(t)})-\bar{m}]^2}$,

$\beta_0^{(t+1)} = \bar{y} - \beta_1^{(t+1)}\bar{m}$,

$\sigma_b^{2(t+1)} = \frac{1}{\sum_{g=1}^{G} n_g}\sum_{g=1}^{G}\sum_{i=1}^{n_g}[m_x(y_{gi},w_{gi};\theta^{(t)}) - \mu_g^{(t+1)}]^2 + v_x(\theta^{(t)})$,

$\sigma_\varepsilon^{2(t+1)} = \frac{1}{\sum_{g=1}^{G} n_g}\sum_{g=1}^{G}\sum_{i=1}^{n_g}[y_{gi} - \beta_0^{(t+1)} - \beta_1^{(t+1)}m_x(y_{gi},w_{gi};\theta^{(t)})]^2 + v_x(\theta^{(t)})$.

If we keep updating estimates by this EM algorithm, then $\theta^{(t)}$ will converge the true MLE of θ. Note that no Monte Carlo method is necessary for the simple linear case.

2) Logistic regression

Parameters in the logistic regression model are $\theta_1 = (\beta_0, \beta_1)$, $\theta_2 = \sigma_\eta^2$ and $\theta_3 = (\mu, \sigma_b^2)$. As in the simple linear case, σ_η^2 is assumed to be known. The E step for this model gives

$$\begin{aligned} Q(\theta|\theta^{(t)}) &= E_{\theta^{(t)}}[l_c(\theta;\mathbf{Y},\mathbf{W},\mathbf{X})|\mathbf{y},\mathbf{w}] \\ &= -\frac{1}{2}\left(\sum_{g=1}^{G} n_g\right)(\ln\sigma_\eta^2 + \ln\sigma_b^2) \\ &+ \sum_{g=1}^{G}\sum_{i=1}^{n_g}\{y_{gi}E_{\theta^{(t)}}[\ln p(X_{gi};\beta)|y_{gi},w_{gi}] + (1-y_{gi})E_{\theta^{(t)}}[\ln(1-p(X_{gi};\beta))|y_{gi},w_{gi}]\} \\ &- \frac{1}{2\sigma_\eta^2}\sum_{g=1}^{G}\sum_{i=1}^{n_g}E_{\theta^{(t)}}[(W_{gi}-X_{gi})^2|y_{gi},w_{gi}] - \frac{1}{2\sigma_b^2}\sum_{g=1}^{G}\sum_{i=1}^{n_g}E_{\theta^{(t)}}[(X_{gi}-\mu_g)^2|y_{gi},w_{gi}] \end{aligned} \quad (2)$$

In fact, the third term of $Q(\theta|\theta^{(t)})$ is constant because σ_η^2 is known. Since the conditional density of X_{gi} given $Y_{gi} = y_{gi}$ and $W_{gi} = w_{gi}$ is

$$f(x_{gi}|y_{gi},w_{gi};\theta) = \frac{p(x_{gi};\beta)^{y_{gi}}[1-p(x_{gi};\beta)]^{1-y_{gi}}h(x_{gi};w_{gi},\mu_i,\sigma_\eta^2,\sigma_b^2)}{\int p(x_{gi};\beta)^{y_{gi}}[1-p(x_{gi};\beta)]^{1-y_{gi}}h(x_{gi};w_{gi},\mu_i,\sigma_\eta^2,\sigma_b^2)dx_{gi}}$$

where $h(x_{gi};w_{gi},\mu_i,\sigma_b^2,\sigma_{\eta^2})$ is the p.d.f of $N(\frac{\sigma_b^2 w_{gi}+\sigma_\eta^2\mu_i}{\sigma_b^2+\sigma_\eta^2}, \frac{\sigma_b^2\sigma_\eta^2}{\sigma_b^2+\sigma_{\eta^2}})$, conditional expectations in $Q(\theta|\theta^{(t)})$ do not have closed form of expressions. Thus, a Monte-Carlo EM method is used as is generally the case in many similar situations. The outline of the M-Step in the $(t+1)$st iteration of the EM algorithm can be described as follows:

Step 1: Set $\mu^{(t+1)}$ equal to the isotonic regression of $(\bar{m}_1,\cdots,\bar{m}_G)'$ with weight vector $(n_1,\cdots,n_G)'$, where $\bar{m}_g = \frac{1}{n_g}\sum_{i=1}^{n_g}E_{\theta^{(t)}}[X_{gi}|y_{gi},w_{gi}]$. Then, compute $\sigma_b^{2(t+1)} = \frac{1}{\sum_{g=1}^{G} n_g}\sum_{g=1}^{G}\sum_{i=1}^{n_g}E_{\theta^{(t)}}[(X_{gi}-\mu^{(t+1)})^2|y_{gi},w_{gi}]$, similarly.

Step 2: Keeping $\theta^{(t)}$ in the conditional distribution, apply a usual Newton method to maximize $Q(\theta|\theta^{(t)})$ with respect to β until a convergence criterion is satisfied. And set $\beta^{(t+1)}$ equal to the solution.

It should be noted that the Newton method in Step 2 can be applied simply to the second term in $Q(\theta|\theta^{(t)})$ because all other conditional expectations do not involve β.

EM with Measurement Errors and Missing in Covariate:

1) Linear regression

In this case, $Q_1(\theta|\theta^{(t)})$ is the same as (4) while $Q_2(\theta|\theta^{(t)})$ is given by

$$\begin{aligned} Q_2(\theta|\theta^{(t)}) &= -\frac{1}{2}\left(\sum_{g=1}^{G} n_g^*\right)(\ln\sigma_\eta^2+\ln\sigma_b^2+\ln\sigma_\varepsilon^2) - \frac{1}{2\sigma_\varepsilon^2}\sum_{g=1}^{G}\sum_{i=1}^{n_g^*}E_{\theta^{(t)}}[(Y_{gi}^*-\beta_0-\beta_1X_{gi}^*)^2|y_{gi}^*] \\ &\quad -\frac{1}{2\sigma_\eta^2}\sum_{g=1}^{G}\sum_{i=1}^{n_g^*}E_{\theta^{(t)}}[(W_{gi}^*-X_{gi}^*)^2|y_{gi}^*] - \frac{1}{2\sigma_b^2}\sum_{g=1}^{G}\sum_{i=1}^{n_g^*}E_{\theta^{(t)}}[(X_{gi}^*-\mu_g)^2|y_{gi}^*]. \end{aligned} \tag{3}$$

Recall first $(X_{gi}|y_{gi},w_{gi})$ follows $N(m_x(y_{gi},w_{gi};\theta),v_x(\theta))$. Also note that $(X_{gi}^*,W_{gi}^*|y_{gi}^*)$ follows a bivariate normal distribution $BVN(m_{x^*}(y_{gi}^*;\theta),m_{w^*}(y_{gi}^*;\theta),\rho_{x^*w^*}(\theta),v_{x^*}(\theta),v_{w^*}(\theta))$ where $m_{x^*}(y_{gi}^*;\theta)=m_{w^*}(y_{gi}^*;\theta)=\frac{\sigma_\varepsilon^2\mu_g+\beta_1\sigma_b^2(y_{gi}^*-\beta_0)}{\sigma_\varepsilon^2+\beta_1^2\sigma_b^2}$, $\rho_{x^*w^*}(\theta)=[\frac{\sigma_\varepsilon^2\sigma_b^2}{\sigma_\varepsilon^2\sigma_b^2+\sigma_\varepsilon^2\sigma_{\eta^2}+\beta_1^2\sigma_b^2\sigma_\eta^2}]^{\frac{1}{2}}$, $v_{x^*}(\theta)=\frac{\sigma_\varepsilon^2\sigma_b^2}{\sigma_\varepsilon^2+\beta_1^2\sigma_b^2}$, and $v_{w^*}(\theta)=\frac{\sigma_\varepsilon^2\sigma_b^2+\sigma_\varepsilon^2\sigma_{\eta^2}+\beta_1^2\sigma_b^2\sigma_\eta^2}{\sigma_\varepsilon^2+\beta_1^2\sigma_b^2}$. Similarly to the case without missing, let $\overline{m}_g=\frac{1}{n_g+n_g^*}\sum_{i=1}^{n_g}[m_x(y_{gi},w_{gi};\theta^{(t)})+m_{x^*}(y_{gi}^*;\theta^{(t)})]$, $g=1,\cdots,G$, $\overline{m}=\frac{1}{\sum_{g=1}^{G}(n_g+n_g^*)}\sum_{g=1}^{G}(n_g+n_g^*)\bar{m}_g$, and $\overline{y}=\frac{1}{\sum_{g=1}^{G}(n_g+n_g^*)}\sum_{g=1}^{G}(\sum_{i=1}^{n_g}y_{gi}+\sum_{i=1}^{n_g^*}y_{gi}^*)$. Then, considering (1) and (3), we can establish the EM algorithm that updates estimates as follows:

$$\begin{aligned} \mu^{(t+1)} &= \text{isotonic regression of } (\bar{m}_1,\bar{m}_2,\cdots,\bar{m}_G)' \text{ with weight vector } (n_1+n_1^*,\ n_2+n_2^*,\ \cdots,\ n_G+n_G^*)', \\ \beta_1^{(t+1)} &= \frac{\sum_{g=1}^{G}\{\sum_{i=1}^{n_g}[m_x(y_{gi},w_{gi};\theta^{(t)})-\bar{m}]y_{gi}+\sum_{i=1}^{n_g^*}[m_{x^*}(y_{gi}^*;\theta^{(t)})-\bar{m}]y_{gi}^*\}}{\sum_{g=1}^{G}\{\sum_{i=1}^{n_g}[m_x(y_{gi},w_{gi};\theta^{(t)})-\bar{m}]^2+\sum_{i=1}^{n_g^*}[m_{x^*}(y_{gi}^*;\theta^{(t)})-\bar{m}]^2\}}, \\ \beta_0^{(t+1)} &= \bar{y}-\beta_1^{(t+1)}\bar{m}, \\ \sigma_b^{2(t+1)} &= \frac{1}{\sum_{g=1}^{G}(n_g+n_g^*)}\sum_{g=1}^{G}\left\{\sum_{i=1}^{n_g}[m_x(y_{gi},w_{gi};\theta^{(t)})-\mu_g^{(t+1)}]^2+[m_{x^*}(y_{gi}^*;\theta^{(t)})-\mu_g^{(t+1)}]^2\right. \\ &\quad \left. +n_gv_x(\theta^{(t)})+n_g^*v_{x^*}(\theta^{(t)})\right\}, \\ \sigma_\varepsilon^{2(t+1)} &= \frac{1}{\sum_{g=1}^{G}(n_g+n_g^*)}\sum_{g=1}^{G}\left\{\sum_{i=1}^{n_g}[y_{gi}-\beta_0^{(t+1)}-\beta_1^{(t+1)}m_x(y_{gi},w_{gi};\theta^{(t)})]^2\right. \\ &\quad \left. +\sum_{i=1}^{n_g^*}[y_{gi}^*-\beta_0^{(t+1)}-\beta_1^{(t+1)}m_{x^*}(y_{gi}^*;\theta^{(t)})]^2+n_gv_x(\theta^{(t)})+n_g^*v_{x^*}(\theta^{(t)})\right\}. \end{aligned}$$

2) Logistic regression

Based on observations having missing values in covariate, the second term of $Q(\theta|\theta^{(t)})$ for this model is

expressed as

$$
\begin{aligned}
Q_2(\theta|\theta^{(t)}) &= -\frac{1}{2}\left(\sum_{g=1}^{G} n_g\right)(\ln\sigma_\eta^2 + \ln\sigma_b^2) \\
&+ \sum_{g=1}^{G}\sum_{i=1}^{n_g^*}\{y_{gi}^* E_{\theta^{(t)}}[\ln p(X_{gi}^*;\beta)|y_{gi}^*] + (1-y_{gi}^*)E_{\theta^{(t)}}[\ln(1-p(X_{gi}^*;\beta))|y_{gi}^*]\} \\
&- \frac{1}{2\sigma_\eta^2}\sum_{g=1}^{G}\sum_{i=1}^{n_g^*} E_{\theta^{(t)}}[(W_{gi}^* - X_{gi}^*)^2|y_{gi}^*] - \frac{1}{2\sigma_b^2}\sum_{g=1}^{G}\sum_{i=1}^{n_g^*} E_{\theta^{(t)}}[(X_{gi}^* - \mu_g)^2|y_{gi}^*]. \qquad (4)
\end{aligned}
$$

In order to maximize $Q(\theta|\theta^{(t)})$, we need a Newton method as a part of each EM procedure. However, our investigations indicate that it does not take too long time to reach a convergence criterion. Considering (2) and (4), the M-Step can be summarized as follows:

Step 1: Set $\mu^{(t+1)}$ equal to the isotonic regression of $(\bar{m}_1, \cdots, \bar{m}_G)'$ with weight vector $(n_1 + n_1^*, \cdots, n_G + n_G^*)'$, where $\bar{m}_g = \frac{1}{n_g + n_g^*}\{\sum_{i=1}^{n_g} E_{\theta^{(t)}}[X_{gi}|y_{gi}, w_{gi}] + \sum_{i=1}^{n_g^*} E_{\theta^{(t)}}[X_{gi}^*|y_{gi}^*]\}$. Then, compute $\sigma_b^{2(t+1)} = \frac{1}{\sum_{g=1}^{G}(n_g + n_g^*)}\sum_{g=1}^{G}\{\sum_{i=1}^{n_g} E_{\theta^{(t)}}[(X_{gi} - \mu^{(t+1)})^2|y_{gi}, w_{gi}] + \sum_{i=1}^{n_g^*} E_{\theta^{(t)}}[(X_{gi}^* - \mu^{(t+1)})^2|y_{gi}^*]\}$, similarly.

Step 2: Keeping $\theta^{(t)}$ in the conditional distributions, plug $\mu^{(t+1)}$ and $\sigma_b^{2(t+1)}$ into $Q(\theta|\theta^{(t)})$ and apply a usual Newton method to maximize $Q(\theta|\theta^{(t)})$ with respect to β. Set $\beta^{(t+1)}$ equal to the solution.

As mentioned earlier, the conditional expectations here do not have closed form of expressions, and thus we rely on a Monte Carlo method to evaluate them.
