# Supplementary material for: Estimation methods with ordered exposure subject to measurement error and missingness in semi-ecological design
Source: BMC Med Res Methodol. 2012 Sep 4;12:135. doi: 10.1186/1471-2288-12-135 (PMC3552934; doi:10.1186/1471-2288-12-135)
Supplement: Additional file 2 — Tables from Simulation Studies. Table S1. Empirical bias of β^1 (with empirical MSE in parentheses) in the linear model when between-group contrast is 0.3. Table S2. Empirical bias of β^1 (with empirical MSE in parentheses) in the linear model when between-group contrast is 0.5. Table S3. Empirical bias of β^1 (with empirical MSE in parentheses) in the logistic model when between-group contrast is 0.3. Table S4. Empirical bias of β^1 (with empirical MSE in parentheses) in the logistic model when between-group contrast is 0.5. [file 1471-2288-12-135-S2.pdf]

Table 1: Empirical **bias** of $\hat{\beta}_1$ (with empirical **MSE** in parentheses) in the linear model when between-group contrast is 0.3.

$\delta = 0.3$	σ_b^2	σ_η^2	Naive	GBS	CGBS	CEM
With full values ($n_y = 30$ and $n_x = 30$)						
	0.3	0.25	0.356(0.020)	0.025(0.030)	0.023(0.031)	0.015(0.020)
		0.50	0.522(0.031)	0.041(0.031)	0.033(0.032)	0.012(0.025)
		0.75	0.621(0.040)	0.057(0.033)	0.038(0.034)	0.012(0.028)
	0.6	0.25	0.255(0.012)	0.026(0.029)	0.020(0.030)	0.011(0.011)
		0.50	0.402(0.020)	0.042(0.030)	0.030(0.031)	0.009(0.014)
		0.75	0.501(0.027)	0.062(0.031)	0.038(0.033)	0.005(0.018)
	0.9	0.25	0.198(0.009)	0.027(0.028)	0.019(0.030)	0.009(0.008)
		0.50	0.327(0.014)	0.045(0.029)	0.030(0.031)	0.008(0.010)
		0.75	0.420(0.020)	0.072(0.032)	0.038(0.033)	0.005(0.012)
With missing values ($n_y = 30$ and $n_x = 20$)						
	0.3	0.25	–	0.012(0.043)	0.003(0.044)	0.021(0.032)
		0.50	–	0.028(0.047)	0.003(0.052)	0.035(0.042)
		0.75	–	0.057(0.048)	0.005(0.067)	0.045(0.048)
	0.6	0.25	–	0.024(0.044)	0.008(0.046)	0.015(0.018)
		0.50	–	0.048(0.047)	0.001(0.060)	0.029(0.026)
		0.75	–	0.071(0.053)	0.018(0.092)	0.041(0.033)
	0.9	0.25	–	0.039(0.043)	0.011(0.049)	0.012(0.011)
		0.50	–	0.061(0.049)	0.004(0.071)	0.023(0.016)
		0.75	–	0.078(0.060)	0.040(0.143)	0.035(0.021)
With missing values ($n_y = 30$ and $n_x = 10$)						
	0.3	0.25	–	0.058(0.046)	0.021(0.046)	0.064(0.050)
		0.50	–	0.113(0.048)	0.031(0.052)	0.079(0.075)
		0.75	–	0.169(0.049)	0.033(0.061)	0.089(0.083)
	0.6	0.25	–	0.092(0.045)	0.023(0.050)	0.018(0.035)
		0.50	–	0.147(0.047)	0.028(0.058)	0.051(0.052)
		0.75	–	0.204(0.049)	0.030(0.066)	0.079(0.062)
	0.9	0.25	–	0.132(0.044)	0.022(0.054)	0.003(0.026)
		0.50	–	0.184(0.046)	0.026(0.061)	0.028(0.036)
		0.75	–	0.239(0.050)	0.021(0.077)	0.053(0.046)

Empirical **bias** of $\hat{\beta}_1$ (with empirical **MSE** in parentheses) in 200 simulations when the disease model is **linear**, estimated by different methods: naive, group-based strategy (GBS), constrained group-based strategy (CGBS), and Monte Carlo EM(CEM). G: number of groups; n_y: number of outcomes in each group; n_x: number of exposures measured; δ: between-group contrast; σ_b^2: between-subject variability; σ_η^2: measurement-error variability; bias = $\frac{|\hat{\beta}_1 - \beta_1|}{\beta_1}$; MSE=$E[(\hat{\beta}_1 - \beta_1)^2]$.

Table 2: Empirical **bias** of $\hat{\beta}_1$ (with empirical **MSE** in parentheses) in the linear model when between-group contrast is 0.5.

$\delta = 0.5$	σ_b^2	σ_η^2	Naive	GBS	CGBS	CEM
With full values ($n_y = 30$ and $n_x = 30$)						
	0.3	0.25	0.249(0.012)	0.013(0.012)	0.013(0.012)	0.012(0.010)
		0.50	0.395(0.019)	0.020(0.012)	0.019(0.012)	0.010(0.011)
		0.75	0.493(0.026)	0.026(0.012)	0.024(0.012)	0.008(0.012)
	0.6	0.25	0.195(0.008)	0.013(0.011)	0.013(0.011)	0.011(0.007)
		0.50	0.323(0.013)	0.020(0.012)	0.018(0.012)	0.010(0.008)
		0.75	0.415(0.019)	0.026(0.012)	0.023(0.012)	0.009(0.009)
	0.9	0.25	0.161(0.006)	0.014(0.011)	0.013(0.011)	0.009(0.006)
		0.50	0.273(0.010)	0.019(0.011)	0.018(0.012)	0.009(0.006)
		0.75	0.358(0.015)	0.026(0.012)	0.022(0.012)	0.009(0.007)
With missing values ($n_y = 30$ and $n_x = 20$)						
	0.3	0.25	–	0.002(0.014)	0.002(0.014)	0.012(0.013)
		0.50	–	0.007(0.015)	0.005(0.015)	0.019(0.015)
		0.75	–	0.012(0.017)	0.008(0.017)	0.026(0.017)
	0.6	0.25	–	0.006(0.014)	0.005(0.014)	0.012(0.010)
		0.50	–	0.011(0.016)	0.007(0.016)	0.020(0.013)
		0.75	–	0.015(0.018)	0.009(0.018)	0.027(0.015)
	0.9	0.25	–	0.010(0.015)	0.007(0.015)	0.011(0.008)
		0.50	–	0.015(0.017)	0.009(0.017)	0.018(0.010)
		0.75	–	0.019(0.018)	0.010(0.019)	0.025(0.012)
With missing values ($n_y = 30$ and $n_x = 10$)						
	0.3	0.25	–	0.017(0.017)	0.010(0.017)	0.034(0.018)
		0.50	–	0.037(0.018)	0.020(0.018)	0.054(0.021)
		0.75	–	0.057(0.019)	0.029(0.019)	0.069(0.025)
	0.6	0.25	–	0.028(0.017)	0.016(0.017)	0.028(0.016)
		0.50	–	0.048(0.019)	0.023(0.018)	0.048(0.020)
		0.75	–	0.068(0.020)	0.030(0.020)	0.066(0.024)
	0.9	0.25	–	0.040(0.018)	0.021(0.018)	0.020(0.013)
		0.50	–	0.060(0.019)	0.027(0.019)	0.037(0.017)
		0.75	–	0.080(0.020)	0.032(0.021)	0.053(0.021)

Empirical **bias** of $\hat{\beta}_1$ (with empirical **MSE** in parentheses) in 200 simulations when the disease model is **linear**, estimated by different methods: naive, group-based strategy (GBS), constrained group-based strategy (CGBS), and Monte Carlo EM(CEM). G: number of groups; n_y: number of outcomes in each group; n_x: number of exposures measured; δ: between-group contrast; σ_b^2: between-subject variability; σ_η^2: measurement-error variability; bias = $\frac{|\hat{\beta}_1 - \beta_1|}{\beta_1}$; MSE=$E[(\hat{\beta}_1 - \beta_1)^2]$.

Table 3: Empirical **bias** of $\hat{\beta}_1$ (with empirical **MSE** in parentheses) in the logistic model when between-group contrast is 0.3.

$\delta = 0.3$	σ_b^2	σ_η^2	Naive	GBS	CGBS	CEM
With full values ($n_y = 30$ and $n_x = 30$)						
	0.3	0.25	0.381(0.091)	0.134(0.315)	0.137(0.316)	0.018(0.194)
		0.50	0.549(0.087)	0.112(0.317)	0.125(0.320)	0.038(0.234)
		0.75	0.646(0.086)	0.086(0.325)	0.114(0.329)	0.057(0.262)
	0.6	0.25	0.284(0.064)	0.167(0.313)	0.174(0.315)	0.010(0.103)
		0.50	0.439(0.063)	0.138(0.318)	0.159(0.320)	0.005(0.144)
		0.75	0.542(0.064)	0.108(0.330)	0.147(0.330)	0.023(0.183)
	0.9	0.25	0.229(0.047)	0.151(0.303)	0.157(0.307)	0.028(0.067)
		0.50	0.366(0.048)	0.124(0.308)	0.142(0.313)	0.038(0.088)
		0.75	0.463(0.050)	0.097(0.318)	0.128(0.322)	0.030(0.108)
With missing values ($n_y = 30$ and $n_x = 20$)						
	0.3	0.25	–	0.200(0.299)	0.182(0.302)	0.077(0.224)
		0.50	–	0.224(0.295)	0.195(0.302)	0.107(0.297)
		0.75	–	0.244(0.292)	0.206(0.303)	0.126(0.325)
	0.6	0.25	–	0.270(0.288)	0.242(0.301)	0.071(0.128)
		0.50	–	0.297(0.280)	0.259(0.300)	0.073(0.173)
		0.75	–	0.322(0.272)	0.274(0.299)	0.078(0.223)
	0.9	0.25	–	0.299(0.294)	0.280(0.317)	0.056(0.094)
		0.50	–	0.323(0.283)	0.295(0.318)	0.045(0.119)
		0.75	–	0.347(0.274)	0.310(0.319)	0.048(0.153)
With missing values ($n_y = 30$ and $n_x = 10$)						
	0.3	0.25	–	0.095(0.353)	0.025(0.345)	0.136(0.488)
		0.50	–	0.142(0.357)	0.035(0.349)	0.124(0.588)
		0.75	–	0.180(0.354)	0.050(0.357)	0.032(0.763)
	0.6	0.25	–	0.158(0.373)	0.031(0.370)	0.181(0.375)
		0.50	–	0.219(0.368)	0.051(0.378)	0.231(0.454)
		0.75	–	0.251(0.343)	0.057(0.386)	0.273(0.636)
	0.9	0.25	–	0.195(0.360)	0.002(0.396)	0.143(0.303)
		0.50	–	0.269(0.363)	0.024(0.421)	0.221(0.365)
		0.75	–	0.298(0.323)	0.020(0.432)	0.267(0.570)

Empirical **bias** of $\hat{\beta}_1$ (with empirical **MSE** in parentheses) in 200 simulations when the disease model is **logistic**, estimated by different methods: naive, group-based strategy (GBS), constrained group-based strategy (CGBS), and Monte Carlo EM(CEM). G: number of groups; n_y: number of outcomes in each group; n_x: number of exposures measured; δ: between-group contrast; σ_b^2: between-subject variability; σ_η^2: measurement-error variability; bias = $\frac{|\hat{\beta}_1 - \beta_1|}{\beta_1}$; MSE=$E[(\hat{\beta}_1 - \beta_1)^2]$.

Table 4: Empirical **bias** of $\hat{\beta}_1$ (with empirical **MSE** in parentheses) in the logistic model when between-group contrast is 0.5.

$\delta = 0.5$	σ_b^2	σ_η^2	Naive	GBS	CGBS	CEM
With full values ($n_y = 30$ and $n_x = 30$)						
	0.3	0.25	0.278(0.053)	0.068(0.101)	0.068(0.101)	0.006(0.083)
		0.50	0.437(0.053)	0.061(0.101)	0.061(0.101)	0.026(0.092)
		0.75	0.542(0.055)	0.054(0.101)	0.055(0.101)	0.022(0.096)
	0.6	0.25	0.215(0.042)	0.103(0.100)	0.103(0.100)	0.001(0.058)
		0.50	0.349(0.043)	0.095(0.100)	0.096(0.100)	0.007(0.071)
		0.75	0.445(0.045)	0.087(0.100)	0.089(0.100)	0.023(0.079)
	0.9	0.25	0.180(0.034)	0.114(0.107)	0.114(0.108)	0.008(0.044)
		0.50	0.299(0.035)	0.106(0.107)	0.107(0.108)	0.003(0.054)
		0.75	0.388(0.038)	0.097(0.107)	0.101(0.108)	0.002(0.064)
With missing values ($n_y = 30$ and $n_x = 20$)						
	0.3	0.25	–	0.055(0.103)	0.055(0.103)	0.042(0.103)
		0.50	–	0.069(0.102)	0.067(0.103)	0.043(0.115)
		0.75	–	0.082(0.102)	0.075(0.103)	0.047(0.119)
	0.6	0.25	–	0.110(0.103)	0.107(0.103)	0.055(0.081)
		0.50	–	0.124(0.102)	0.118(0.103)	0.055(0.093)
		0.75	–	0.138(0.102)	0.125(0.103)	0.056(0.105)
	0.9	0.25	–	0.157(0.101)	0.150(0.101)	0.055(0.063)
		0.50	–	0.170(0.100)	0.159(0.100)	0.051(0.072)
		0.75	–	0.181(0.100)	0.166(0.101)	0.054(0.081)
With missing values ($n_y = 30$ and $n_x = 10$)						
	0.3	0.25	–	0.049(0.113)	0.039(0.112)	0.059(0.182)
		0.50	–	0.072(0.115)	0.051(0.113)	0.044(0.212)
		0.75	–	0.093(0.117)	0.058(0.115)	0.037(0.225)
	0.6	0.25	–	0.078(0.110)	0.051(0.109)	0.089(0.161)
		0.50	–	0.101(0.112)	0.061(0.110)	0.109(0.206)
		0.75	–	0.121(0.114)	0.066(0.112)	0.110(0.219)
	0.9	0.25	–	0.103(0114)	0.063(0.112)	0.087(0.134)
		0.50	–	0.127(0.117)	0.072(0.115)	0.134(0.194)
		0.75	–	0.149(0.118)	0.076(0.118)	0.154(0.239)

Empirical bias of $\hat{\beta}_1$ (with empirical MSE in parentheses) in 200 simulations when the disease model is **logistic**, estimated by different methods: naive, group-based strategy (GBS), constrained group-based strategy (CGBS), and Monte Carlo EM(CEM). G: number of groups; n_y: number of outcomes in each group; n_x: number of exposures measured; δ: between-group contrast; σ_b^2: between-subject variability; σ_η^2: measurement-error variability; bias = $\frac{|\hat{\beta}_1 - \beta_1|}{\beta_1}$; MSE=$E[(\hat{\beta}_1 - \beta_1)^2]$.
